# Supplementary figures and images for: Digital marketing program design based on abnormal consumer behavior data classification and improved homomorphic encryption algorithm
Source: PeerJ Comput Sci. 2023 Nov 29;9:e1690. doi: 10.7717/peerj-cs.1690 (PMC10703031; doi:10.7717/peerj-cs.1690)

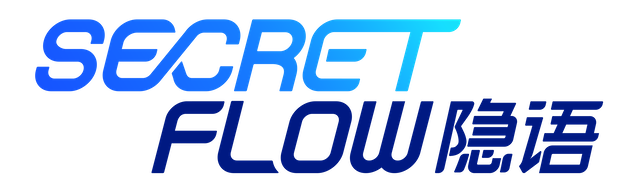

Supplement: Supplemental Information 1 [file peerj-cs-09-1690-s001.zip › code/docs/_static/logo.png]

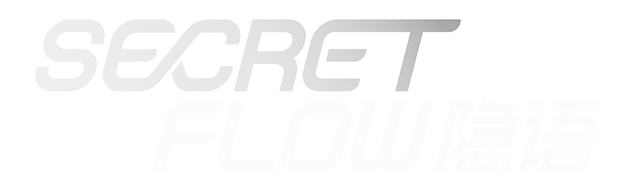

Supplement: Supplemental Information 1 [file peerj-cs-09-1690-s001.zip › code/docs/_static/logo-dark.png]

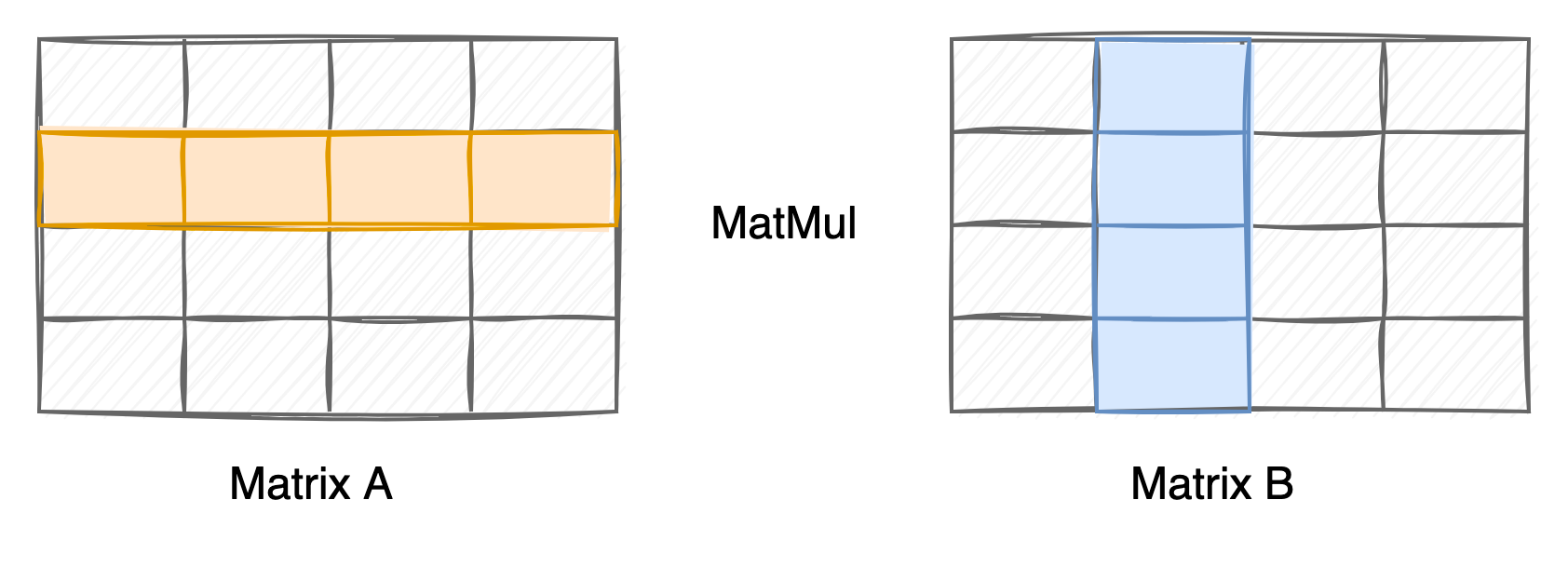

Supplement: Supplemental Information 1 [file peerj-cs-09-1690-s001.zip › code/docs/development/_img/matrix_matmul.png]

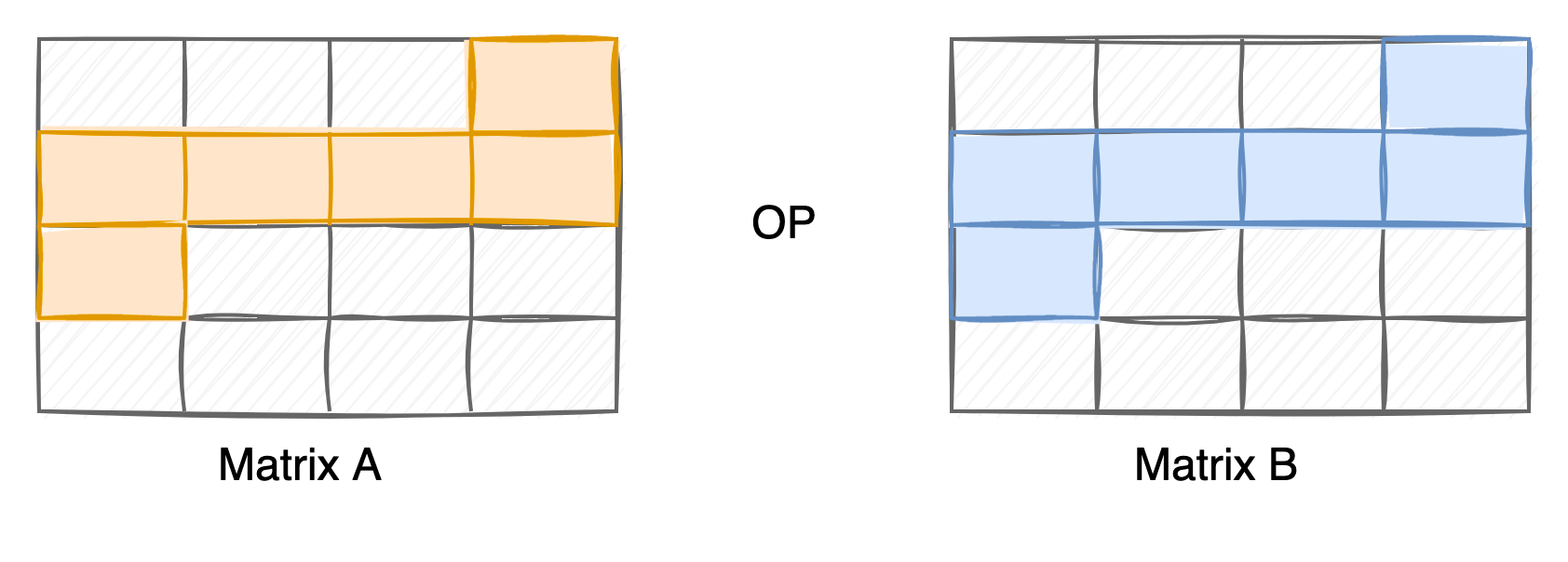

Supplement: Supplemental Information 1 [file peerj-cs-09-1690-s001.zip › code/docs/development/_img/matrix_op.png]

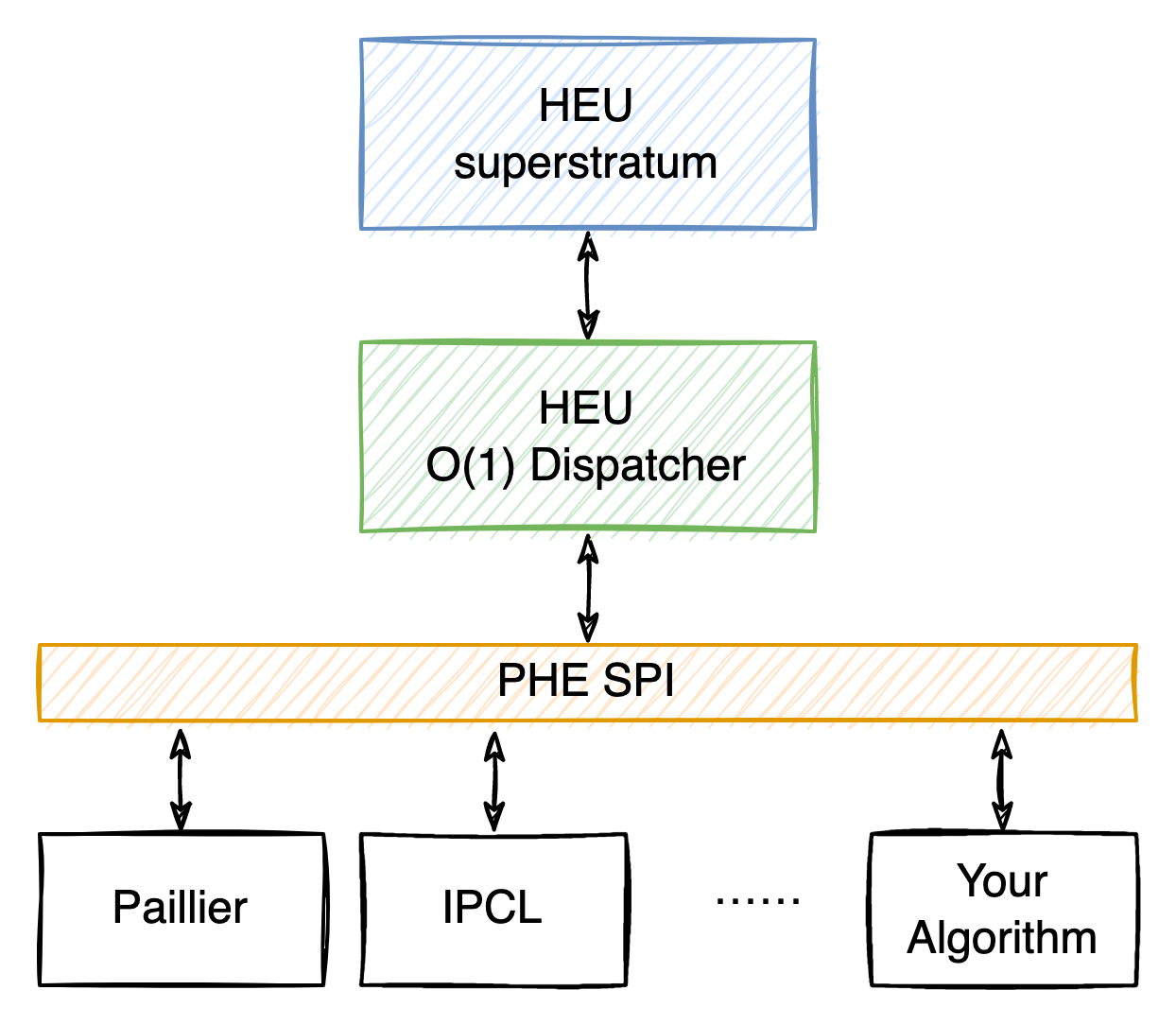

Supplement: Supplemental Information 1 [file peerj-cs-09-1690-s001.zip › code/docs/development/_img/phe_spi.png]

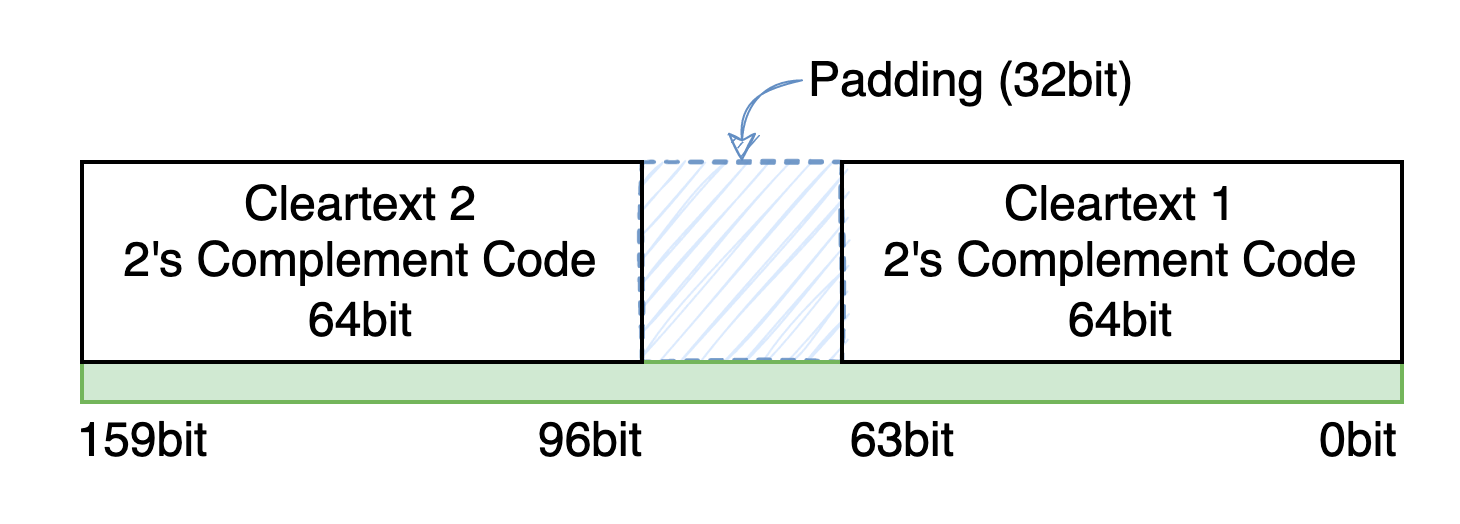

Supplement: Supplemental Information 1 [file peerj-cs-09-1690-s001.zip › code/docs/getting_started/_img/batch_encoding.png]

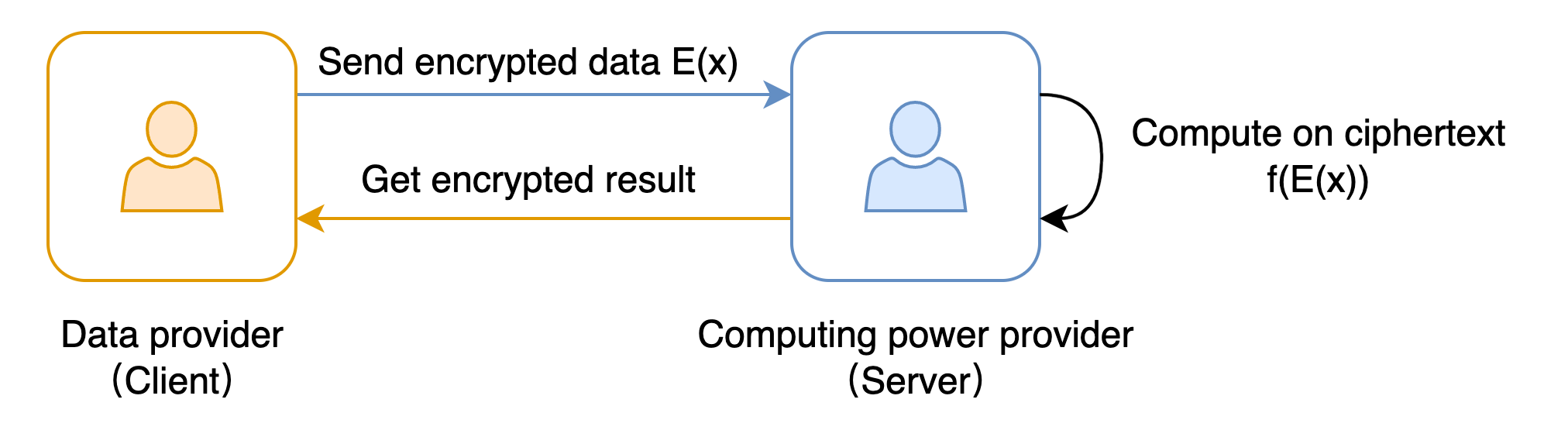

Supplement: Supplemental Information 1 [file peerj-cs-09-1690-s001.zip › code/docs/getting_started/_img/heu_client_server.png]

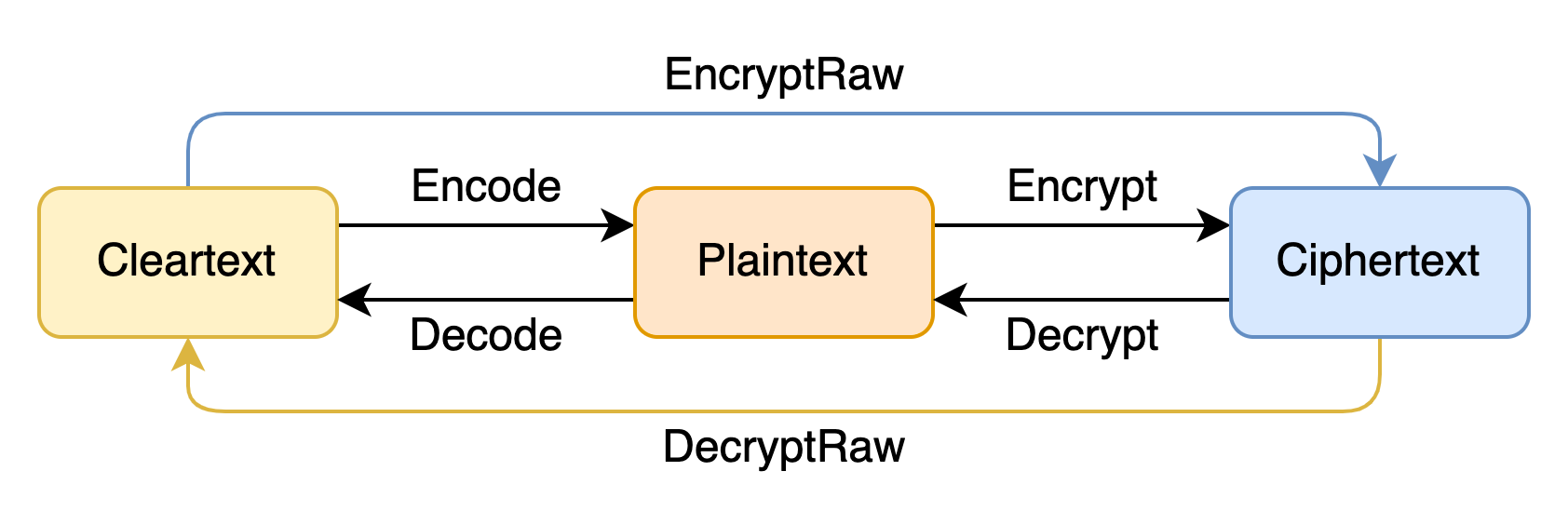

Supplement: Supplemental Information 1 [file peerj-cs-09-1690-s001.zip › code/docs/getting_started/_img/obj_type.png]

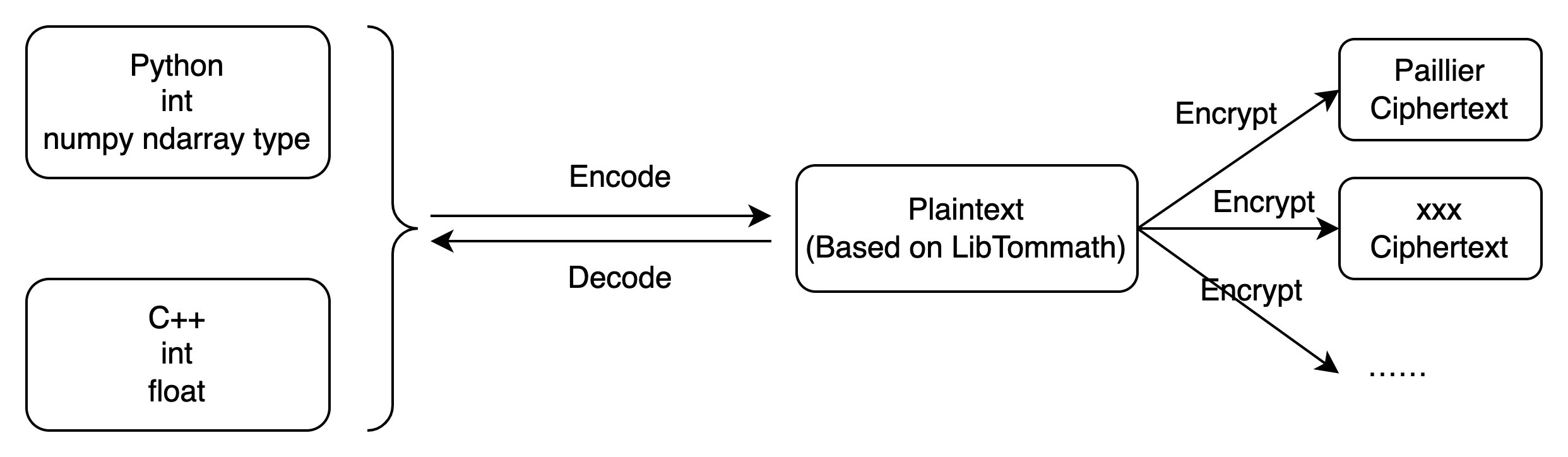

Supplement: Supplemental Information 1 [file peerj-cs-09-1690-s001.zip › code/docs/references/_img/0_2_flow.png]

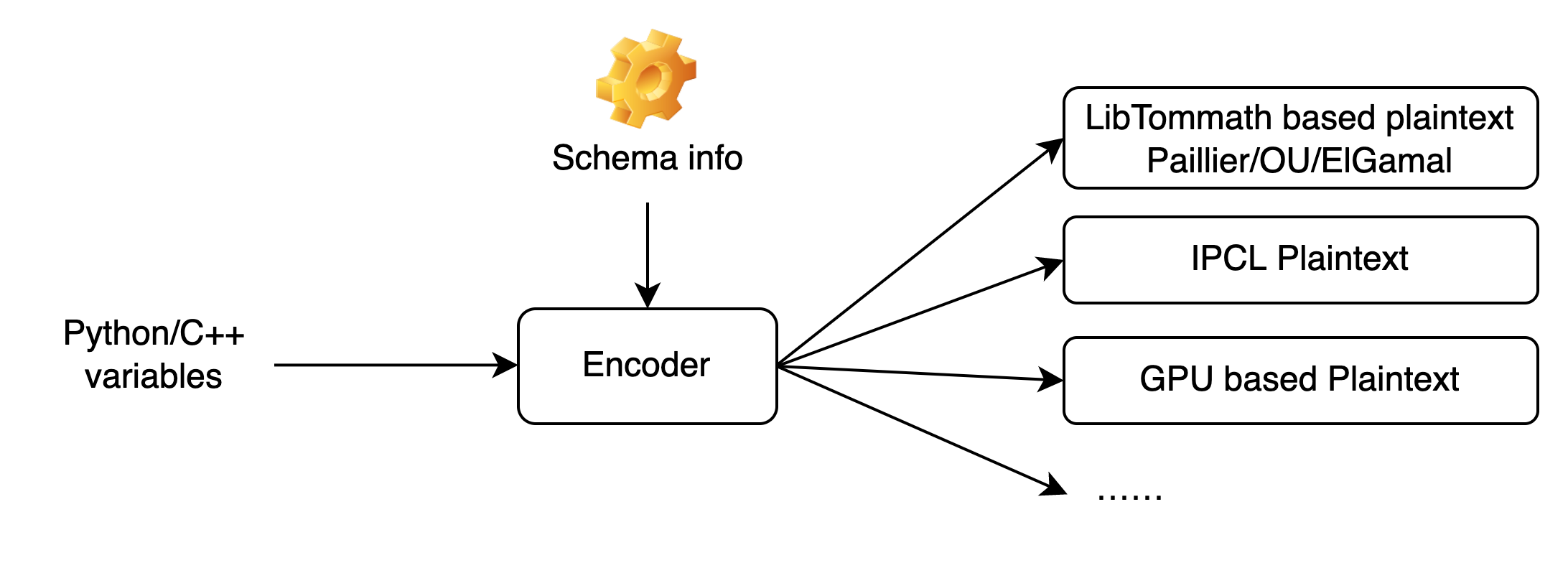

Supplement: Supplemental Information 1 [file peerj-cs-09-1690-s001.zip › code/docs/references/_img/0_3_encoder.png]

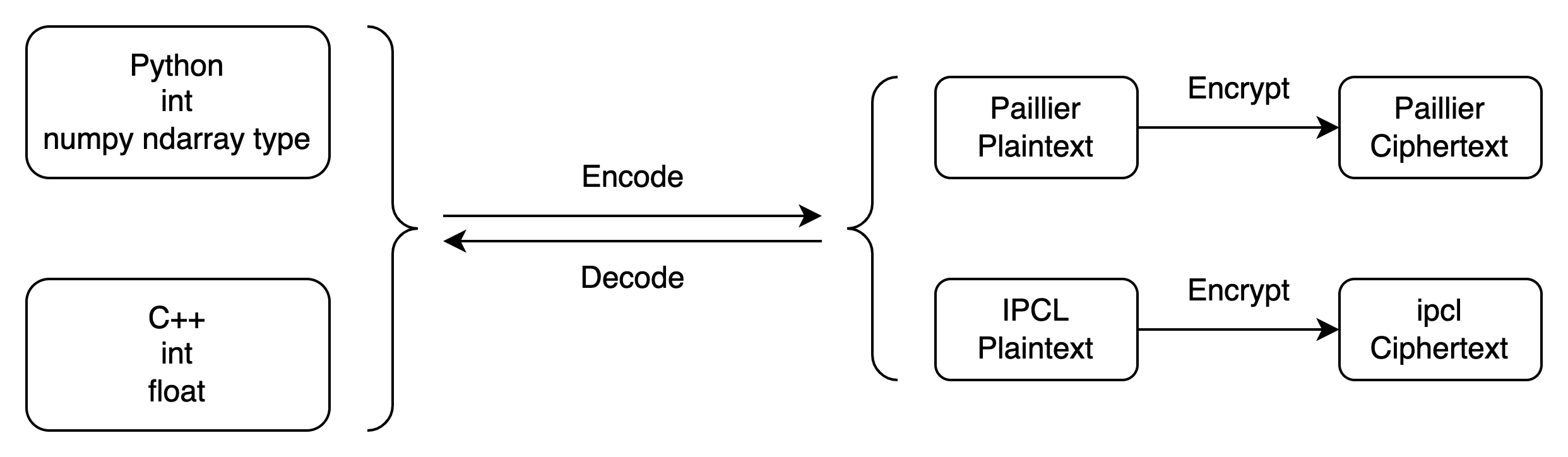

Supplement: Supplemental Information 1 [file peerj-cs-09-1690-s001.zip › code/docs/references/_img/0_3_flow.png]
